# Targeted and untargeted metabolomics and lipidomics in dried blood microsampling: Recent applications and perspectives

**DOI:** 10.1002/ansa.202400002

**Published:** 2024-06-14

**Authors:** Pauline Couacault, Dennisse Avella, Sara Londoño‐Osorio, Ana S. Lorenzo, Ana Gradillas, Olli Kärkkäinen, Elizabeth Want, Michael Witting

**Affiliations:** ^1^ Metabolomics and Proteomics Core Helmholtz Zentrum München Neuherberg Germany; ^2^ Afekta Technologies Ltd. Kuopio Finland; ^3^ School of Pharmacy Faculty of Health Sciences University of Eastern Finland Kuopio Finland; ^4^ Centro de Metabolómica y Bioanálisis (CEMBIO) Facultad de Farmacia Universidad San Pablo‐CEU CEU Universities Urbanización Montepríncipe Boadilla del Monte Madrid Spain; ^5^ Department of Metabolism Digestion and Reproduction Imperial College London London UK; ^6^ Chair of Analytical Food Chemistry TUM School of Life Sciences Technical University of Munich Freising‐Weihenstephan Germany

**Keywords:** blood microsampling, dried blood, LC–MS analysis, lipidomics, metabolomics, targeted and untargeted

## Abstract

Blood microsampling (BµS) offers an alternative to conventional methods that use plasma or serum for profiling human health, being minimally invasive and cost effective, especially beneficial for vulnerable populations. We present a non‐systematic review that offers a synopsis of the analytical methods, applications and perspectives related to dry blood microsampling in targeted and untargeted metabolomics and lipidomics research in the years 2022 and 2023. BµS shows potential in neonatal and paediatric studies, therapeutic drug monitoring, metabolite screening, biomarker research, sports supervision, clinical disorders studies and forensic toxicology. Notably, dried blood spots and volumetric absorptive microsampling options have been more extensively studied than other volumetric technologies. Therefore, we suggest that a further investigation and application of the volumetric technologies will contribute to the use of BµS as an alternative to conventional methods. Conversely, we support the idea that harmonisation of the analytical methods when using BµS would have a positive impact on its implementation.

**Abbreviations**
17‐OHP: 17‐hydroxyprogesterone
21‐OHD: 21‐hydroxylase deficiency
AAS: anabolic androgenic steroids
ACN: acetonitrile
Ala: alanine
Arg: arginine
ASD: autism spectrum disorders
BµS: blood microsampling
CAH: congenital adrenal hyperplasia
DBSs: dried blood spots
DI‐HRMS: direct infusion high‐resolution mass spectrometry
ESI: electrospray ionisation
FA: formic acid
FIA: flow injection analysis
GA1: glutaric aciduria type 1
Gly: glycine
GT: ganglioside
HCT: haematocrit
HESI: heated electrospray ionisation
HILIC: hydrophilic interaction chromatography
HIV: human immunodeficiency virus
HPLC–MS/MS: high‐performance liquid chromatography‐tandem mass spectrometry
IQ: intelligence quotient
ISs: internal standard
LC: liquid chromatography
LLE: liquid–liquid extraction
MACD: multiple acylcarnitine deficiencies
MCADD: medium‐chain acyl‐CoA dehydrogenase deficiency
MeOH: methanol
MMA: methylmalonic acidemia
MRM: multiple reaction monitoring mode
MS: mass spectrometry
MS/MS: tandem mass spectrometry
MTBE: methyl tert‐butyl ether
NBS: newborn screening
Orn: ornithine
OTCD: ornithine transcarbamylase deficiency
PA: phosphatidic acid
PFAS: per‐ and polyfluoroalkyl substances
PGF: prostaglandin F
PGP: phosphatidylglycerolphosphate
Phe: phenylalanine
PIP: phosphatidylinositol phosphate
PIP2: phosphatidylinositol bisphosphate
PKU: phenylketonuria
PMR: parallel reaction monitoring
Q: quadrupole analyser
qDBSs: quantitative dry blood sports
QqQ: triple quadrupole
RP: reverse phase chromatography
SIL: stable isotope labelled
SRM: selective reaction monitoring
TDM: therapeutic drug monitoring
TIMS: trapped ion mobility spectrometry
TOF: time of flight
Tyr: tyrosine
Tyr/Cit: tyrosine/cytosine ratio
UPLC: ultra performance liquid chromatography
Val/Phe: valine/phenylalanine ratio
VAMS: volumetric absorptive microsampling
VBCDs: volumetric blood collection devices
VLCADD: very long‐chain acylcarnitine dehydrogenase deficiency
WADA: World Anti‐Doping Agency

## 1 INTRODUCTION

### 1.1 What is a microsampling technique?

There is an increasing need for alternative specimens for profiling of human health. Traditionally for assessment of health and disease, plasma and serum are used. However, the rather large amount of sample required makes them less useful for repeated sampling in tight intervals or vulnerable groups, such as infants or the elderly. Blood microsampling (BµS) is associated with the collection of typically less than 100 µL of dried or liquid blood samples on absorbing materials. The collection varies depending on the technique, requiring capillary blood droplets from a lancet prick or by capillary/tube absorption.^1^

Dried blood spots (DBSs) have been used in newborn screening (NBS) since 1961 with Robert Guthrie and his test to detect phenylketonuria (PKU) at an early stage in newborn blood.^2^ Nowadays, standard DBS cards are still called Guthrie cards and are commonly used for NBS. In 2022, the USA, Europe and Latin America were the regions with the highest percentage of newborns screened (100, 78 and 32%, respectively).^3^

Compared with venous blood, DBSs are patient‐friendly samples. Indeed, DBS samples only need a small amount of blood (typically < 100 µL)^1^ and are considered minimally invasive. Additionally, DBS collection for most people is not experienced as painful,^4^, ^5^ particularly advantageous for children and people scared of needles. The absence of a need for a phlebotomist or a needle, along with the elimination of centrifugation to separate plasma from blood cells, contributes to the simplicity of the sample collection of those devices. Patients can self‐collect samples at home by pricking one of their fingers and depositing blood drops on the designated card. After a 4‐h drying period, the DBS card can be sent via standard mail to the analysing laboratory. Additionally, the COVID‐19 pandemic highlighted a growing acceptance of home testing and self‐sampling, preventing many individuals from visiting hospitals. Due to the lockdown, the use of self‐sampling methods, such as DBS cards, has increased these last years.^6^ Furthermore, DBS samples are easily stored and show good stability for many metabolites, which is helpful for blood banks.^7^ Since the samples are dried, the risk of biological contamination and infection is low.^1^ To avoid moisture, which can lead to the degradation of analytes, DBS cards need to be stored with desiccant and avoid air.

As venous blood, DBSs can be used for several types of analyses as lipidomic, proteomic and metabolomic, with only one extraction protocol.^8^ DBS was the first BµS device reported to be analysed by metabolomic analysis, but the haematocrit (HCT) affects the size of the spot, the homogeneity of the sample (known as the ‘haematocrit effect’), and hence the reliability of the quantitative analysis.^9^ The HCT is the volume percentage of red blood cells. The lower the HCT, the lower the viscosity: the blood will spread faster through paper fibres and will make the blood spot large, colourless and less homogenous. With a high HCT, the blood spot will be smaller, more intense in colour and more homogenous.^10^, ^11^ Punching disks of the same size for blood spots with varying HCT levels results in different volumes, leading to significant measurement and quantification errors.^11^ On a traditional DBS card, the spot is punched to remove it from the card. Multiple punches can be made in one DBS spot. But due to the haematocrit effect, depending on where the punch is made, the sample may be different. Other BµS technologies that collect fixed volumes have been developed to overcome the DBS limitations; still, studies using DBSs and new technologies evaluate their stability, reliability/accuracy and applicability.^12^, ^13^, ^14^

### 1.2 Other BµS devices

Since DBSs have been shown to be a convenient sampling solution, it has been consistently developing over time. In parallel, different alternatives from BµS have been developed to overcome the aforementioned problems of DBSs. One of the main problems of DBSs, as already discussed above, is the ‘haematocrit effect’.^15^ To have a more homogeneous sample from one DBS, it is possible to use pattern cards, where a drop of blood is applied in the middle of the cross‐shaped pattern and the blood will spread uniformly across the card, creating four replicate punch zone.^16^ The HemaSpot™ HF device from Spot On Sciences^17^ works with the same technology: two or three drops of blood are applied in the middle of the device. The blood will spread uniformly across the height blades, reducing the haematocrit effect between the height samples.

**FIGURE 1:**
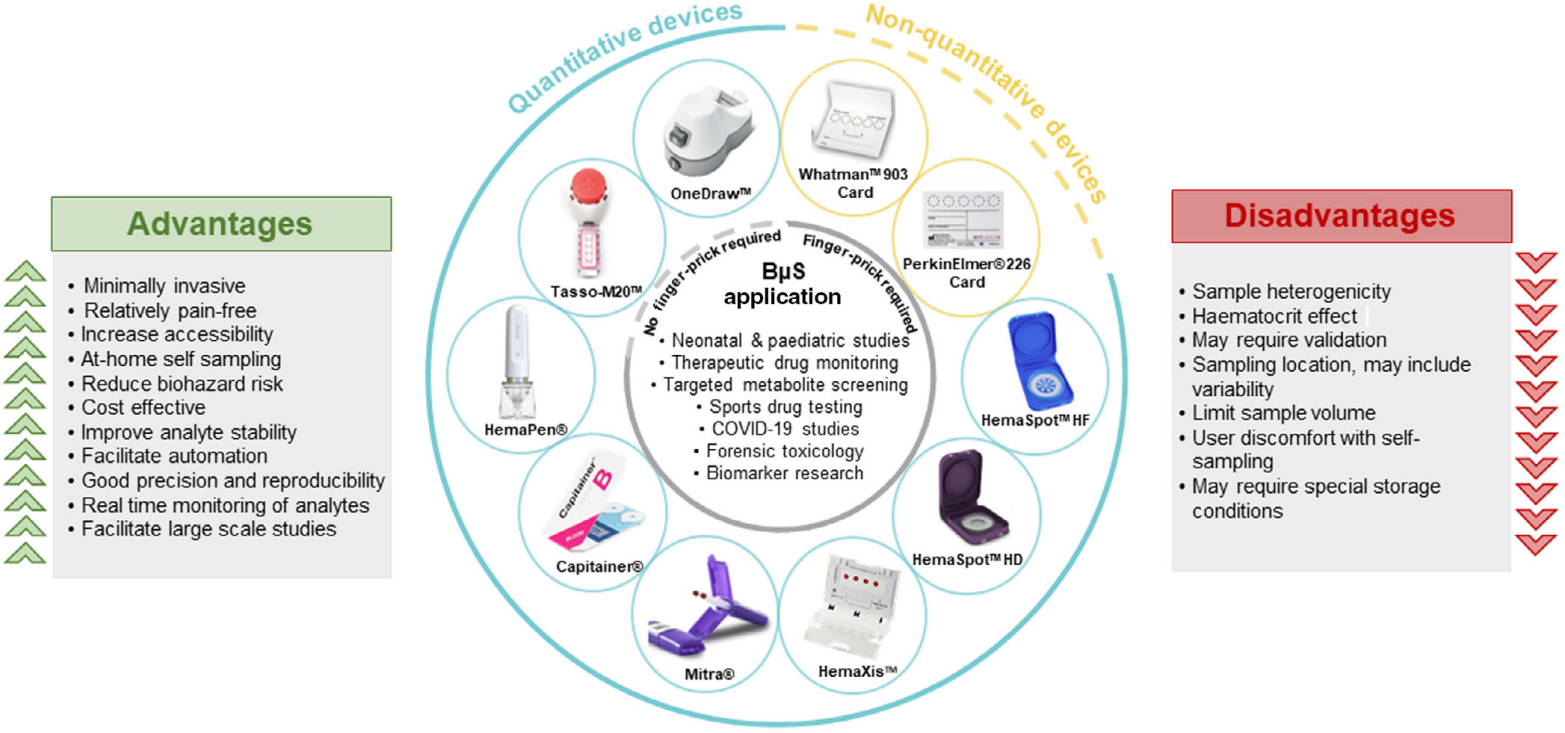
Summary of advantages and disadvantages of blood microsampling (BµS) techniques.

Another solution to overcome the haematocrit effect is the development of quantitative devices to collect an exact volume of capillary blood. The volumetric absorptive microsampling (VAMS)^18^ can absorb a fixed volume (e.g., 10, 20 or 30 µL) of blood depending on the size of its tip via a capillary action. Other devices like qDBS Capitainer^®^,^19^ the hemaPEN^®^ ^20^ or HemaXis™ DB10^21^ have capillary channels to measure a fixed volume of blood, maintaining sample integrity for quantitative analysis. Two additional devices employed for quantitative sample collection, OneDraw™^22^ and Tasso‐M20™, eliminate the need for finger‐piercing, instead opting for application on the upper arm. This approach not only enhances patient acceptability but also minimises discomfort compared with traditional venipuncture and finger‐prick methods.^23^ Figure 1 offers an overview of the frequently used microsampling devices for dried blood collection, and Table 1 provides detailed information about the mechanisms involved in the sample collection process. Instead of creating new collection devices to overcome the haematocrit effect, it is also possible to develop tools to measure the HCT of the blood spot. It can be done optically, with a smartphone app,^11^ reflectance spectroscopy,^24^ near‐infrared or ultraviolet‐visible spectroscopy^25^, ^26^, ^27^ or simply with a scanner and image analysis.^28^

**TABLE 1:** Evolution of BµS devices and their characteristics, based on different advanced technologies.

Sample collection		BµS devices (size/volume)	(Year of introduction)/sample collection description
Finger/heal‐prick required	Traditional dried samples/filter paper card type	DBS Whatman^TM^ 903 Card (5 × 13 mm/75–80 µL)	(1960)/Non‐quantitative device. It is a cellulose cotton‐based filter paper card, where blood drops are spread non‐homogenously via capillary action. https://www.sigmaaldrich.com/ES/es/product/aldrich/wha10534612
		DBS Guthrie cards	(1963)/Non‐quantitative device. It is a cellulose cotton‐based filter paper card, where four blood drops are collected and spread non‐homogenously via capillary action.
		DBS PerkinElmer^®^ 226 Card (5 × 17 mm/100 µL)	(2009)/Non‐quantitative device. It is a cellulose cotton‐based filter paper card, where blood drops are spread non‐homogenously via capillary action. Spots are preperforated in PerkinElmer‐226 Bioanalysis RUO cards. https://resources.perkinelmer.com/lab‐solutions/resources/docs/ifu‐gr226‐226‐sample‐collection‐id1409i‐rev17‐ivd‐eu‐english.pdf
	Advanced dried samples devices	DBS HemaSpot HF device (8 samples from 1 blood drop)	(2010)/Non‐quantitative device. Two or three drops of blood are applied in the middle of the device. The blood will disperse uniformly across the height blades, reducing haematocrit effect between the height samples. https://www.spotonsciences.com/hemaspot‐hf/)
		DBS HemaSpot HD Device (up to 160 µL) (5 drops of blood)	(2010)/Non‐quantitative device. Five drops of blood are applied on the membrane. After one minute, the blood spread on the membrane. A desiccant is placed below the membrane, allowing a quick‐drying. https://www.spotonsciences.com/hemaspot‐hd/
		VAMS Neoteryx Mitra^®^ Device (10, 20 or 30 µL)/CV < 5%	(2016)/Quantitative device. A drop of blood is collected via capillary action to an adsorbent hydrophilic polymeric tip. https://www.neoteryx.com/volumetrically‐accurate‐micro‐sampling‐vams‐collection‐devices)
		qDBS Capitainer^®^ B Collection Device (2 × 10 or 2 × 50 µL)/CV < 5%	(2018)/Quantitative device. For the 10 µL device, a drop of blood is applied to each sample well and the channels measure the right volume. For the 50 µL device, multiple drops of blood are added to the capillary until blood reaches the stop line. When the channels are filled, the blood is transferred onto the pre‐punched Ahlstrom filter paper discs via capillary action. https://capitainer.com/technology/
		DBS hemaPEN^®^ Device (4 × 2.74 ± 0.14 µL)/CV < 2.5%	(2019)/Quantitative device. Blood is collected through four EDTA‐coated capillaries. When they are full, the device is reversed and clicked into its plastic base by the user. The blood is then transferred onto pre‐punched Whatman or Perkin Elmer discs paper via gravity action. https://www.neoteryx.com/hemapen‐dried‐blood‐spot‐collection)
		DBS HemaXis^TM^ DB10 (4 × 10.0 ± 0.5 µL)/CV < 5%	(2020)/Quantitative device. A drop of blood is applied to the inlet of the volumetric capillary channel. When the four channels are full, the device is closed and the blood is transferred to the Whatman or Perkin Elmer paper via capillary action. https://hemaxis.com/products/hemaxis‐db10/)
No‐finger‐prick required		DBS OneDraw™ (2 × 75 µL)	(2019)/Quantitative device. The device is placed onto the skin of the upper arm. When the buttons are pressed, a gentle vacuum and a lancet draws the blood to two paper strips sitting in a cartridge. https://www.drawbridgehealth.com/onedraw/)
		DBS Tasso‐M20™ Device (4 × 17.5 µL)/CV < 5%	(2022)/Quantitative device. The device is placed onto the skin of the upper arm. When the button is pressed, a needle punctures the skin and the blood is drawn onto the sample pods using vacuum. https://www.tassoinc.com/tasso‐m20

Data from manufacturer website as of 7 December, 2023.

### 1.3 Applications of BµS in the targeted and untargeted analysis of small molecules

Many clinical tests rely on the measurement of small molecules (e.g., metabolites, drugs, etc., generally molecules with a mass < 1500 Da), often with single or only a handful of molecules detected simultaneously in one method. Analysis of metabolites is nowadays referred to as metabolomics and can be performed in a targeted manner, focusing on a specific subset of metabolites (e.g., one compound class or metabolic pathway), or untargeted, aiming for comprehensive analysis of as many metabolites as possible. The comprehensive analysis of lipids is called lipidomics and is often regarded as a sub‐discipline of metabolomics. Conceptually, metabolomics can be regarded as an extension of clinical chemistry, analysing more molecules.

Since its initial use in NBS, DBS sampling has evolved to analyse a wide range of endogenous and exogenous compounds. Additionally, untargeted analysis has also been performed. Recent applications of DBS samples include the analysis of small metabolites, such as vitamin D and opioids, as well as larger molecules like peptides and proteins.^29^ VAMS has also seen extensive implementation, particularly in studies related to COVID‐19 analytes, immunosuppressants and anticonvulsants.^30^ For instance, VAMS has been used to study various endogenous metabolites, including creatinine, steroids, vitamins, phosphatidyl ethanols and amino acids, as recently reviewed.^18^ Additionally, advanced emerging technologies such as Capitainer^®^ B‐Vanadate, hemaPEN^®^ and TASSO‐M20™ have shown potential for applications such as caffeine, exercise and steroid monitoring.^31^

Lipidomic analyses follow the same pattern of evolution and development, from the early use of DBSs, for instance, in fatty acid oxidation studies,^32^ to the implementation of quantitative and volumetric analysis with Mitra^®^,^33^ and recently the evaluation of new technologies like hemaPEN^®^ ^34^ and TASSO‐M20™,^35^ in physical exercise studies with athletes and runners. Examples of the classes identified during different studies are fatty acids, polyunsaturated fatty acids, acylcarnitines, sphingolipids, glycerophospholipids, cholesterol, triacylglycerols, prenols, cholesterol esters and sterols.^36^

In 2022, BµS was commonly employed in various biomedical applications, including animal studies, neonatal and paediatric studies, therapeutic drug monitoring (TDM), omics‐based biomarker research and forensic toxicology. Key applications included the development and validation of novel methods, comparison of microsampling with conventional sampling and evaluation of different microsampling devices for optimal application.^1^ However, ongoing research aims to address knowledge gaps in the implementation of microsampling, focusing on preanalytical and analytical conditions, stability's impact on sample metabolites and the potential for self‐sampling and automation.^30^, ^31^ This review focuses on dry BµS studies from 2022 and 2023, specifically liquid chromatography–mass spectrometry (LC–MS) analysis of both targeted and untargeted metabolomics and lipidomics, showcasing the potential benefits of microsampling devices.

## 2 TARGETED METABOLOMICS

### 2.1 Endogenous molecules: endogenous disease biomarkers, NBS

Between 2022 and 2023, eight papers have been published contributing valuable insights into disease applications concerning targeted endogenous metabolite assays in blood samples using various microsampling devices. The focus of these metabolites primarily revolves around amino acids, lipids and small molecules associated with disease markers or NBS.

The majority of those papers adopted DBS collection methods.^37^, ^38^, ^39^, ^40^, ^41^, ^42^ Notably, Gao et al.^43^ deviated from this trend by validating a method using VAMS. In the case of Carling et al.,^44^ a comprehensive comparison of various microsampling devices was conducted, including DBS cards (conventional DBS card (PerkinElmer‐226 filter paper) and PerkinElmer‐226 Bioanalysis RUO cards), volumetric DBSs (HemaXis™ DB10 collection device), VAMS technology (Neoteryx Mitra® device) and quantitative DBSs (qDBS Capitainer® B Collection device). After a qualitative comparison, it was demonstrated that the use of volumetric blood collection devices (VBCDs) in the analysis of phenylalanine (Phe) and tyrosine (Tyr) in blood samples of PKU patients avoids pre‐analytical challenges associated with DBS cards. These challenges include issues related to the volume of blood applied, the HCT effect and the quality of the sample, which had an effect on monitoring the disease.^44^

Concerning sample collection in DBS assays, commonly, a sub‐punch of 3.2 mm in diameter was performed to collect the blood applied,^37^, ^39^, ^40^, ^42^ except for Zhan et al.,^38^ who obtained two 5 mm sub‐punches. In Carling et al.^44^ tests, the sub‐punch in conventional PerkinElmer‐226 filter paper was 8 mm, while in volumetric DBSs (HemaXis™ DB10), it was 7 mm, and in Capitainer^®^, it was 6 mm. The blood volume collected was approximately 20 µL in DBSs from each spot, as it cannot ensure the quantity of volume in each sub‐punch. Gao et al.^43^ collected 20 µL in VAMS devices as well. However, Carling et al.^44^ performed a volume comparison in volumetric and quantitative devices (HemaXis™ DB10, Neoteryx Mitra^®^ and qDBS Capitainer^®^): 20 and 50 µL. The authors demonstrated the lack of a significant volume effect on Phe or Tyr concentrations when using volumetric devices (*p* > 0.01).^44^

During sample extraction, samples were placed in a 96‐well plate or Eppendorf with the extraction solvent mainly using pure methanol,^37^ methanol/water (80:20, v/v)^40^, ^44^ or acetonitrile (ACN)/methanol (1:1, v/v)^43^, ^44^ (others not specifying). This solvent included the labelled internal standard (IS). The following steps involved mixing techniques (e.g., shaking, vortex or sonication at room temperature) and centrifuge for 20 min to 1 h, except for Mak et al.,^40^ Wu et al.^37^ and Gao et al.^43^ which include a dry and reconstitution step with N_2_ gas flow.

The main applications of targeted analysis using microsampling devices are disease biomarkers for diagnosis or monitoring and NBS. Wu et al.^37^ employed a combination of DBS sampling and direct injection mass spectrometry (MS) technology to analyse the blood metabolic profiles of 166 individuals, including gastric cancer patients and healthy controls. Given the limitations of existing low‐sensitivity diagnostic and screening methods for gastric cancer, the identification and selection of highly specific and sensitive biomarkers for early detection and disease monitoring are of critical importance. They identified 23 parameters that were significantly altered in gastric cancer patients compared with healthy individuals. These metabolites were involved in various metabolic pathways, including nucleoside synthesis, glycolysis and lipid and amino acid metabolism, which play crucial roles in cell tumour proliferation and immunosurveillance. A panel of nine metabolites, comprising amino acids (Ala, Arg, Gly, Orn), carnitines (CA‐OH, CAR 10:2) and related ratios (Tyr/Cit, Val/Phe, C5/C3) emerged as an effective tool for discriminating patients with gastric cancer from healthy individuals (AUC: 0.9318) with sensitivity and specificity. These findings suggest that the selected metabolites hold promise as novel biomarkers for gastric cancer detection. In a subsequent step, the authors aim to investigate whether the levels of these metabolites correlate with different stages and severity of gastric cancer.^37^

On the other hand, NBS aims to identify infants with congenital metabolic disorders early in life, enabling timely intervention and improved outcomes. However, current NBS methods have limitations, including false positives and false negatives biomarkers referral rates. Target metabolomics has emerged as a promising tool to overcome these issues associated to NBS.^40^ Zhan et al.^38^ evaluate the effectiveness of steroid profiling in DBSs using liquid chromatography–tandem mass spectrometry (LC–MS/MS) as a second‐tier test for congenital adrenal hyperplasia (CAH) NBS in China. The study analysed DBS samples from newborns with elevated 17‐hydroxyprogesterone (17‐OHP) levels detected by initial immunoassay‐based screening. The steroid profiling by LC–MS/MS was further performed to measure the concentrations of 17‐OHP, 21‐deoxycortisol, progesterone, androstenedione and testosterone. Among 377 newborns with elevated 17‐OHP, steroid profiling confirmed CAH in 171 cases (45.4%). The most common form of CAH was 21‐hydroxylase deficiency (21‐OHD), accounting for 154 cases (90,1%). The positive predictive value of steroid profiling for CAH was 26.0%, significantly higher than that of immunoassay‐based screening alone (15.3%) and the number of false‐positive results reduced by 89.1%, except in premature newborns where it is not ensured. This approach has the potential to improve the accuracy and efficiency of CAH NBS, avoiding extra costs, follow‐up tests and unnecessary family anxiety associated with false positive results. The LC–MS/MS method developed is able to discriminate between the two most frequent forms of CAH, 21‐OHD and 11‐OHD, which helps to reduce the time of the diagnosis. The validation of the LC/MS–MS method for the quantification of steroid hormones in DBS samples showed that the method is robust, reliable and sensitive by evaluating the intra‐day and inter‐day precision, linearity, limit of quantification and recovery. These results indicate that the method is suitable for routine NBS for CAH.^38^

Carling et al.^44^ performed for the first time an evaluation of the accuracy, precision and effect of blood volume and HCT on the measurement of Phe and Tyr to improve monitoring of patients with PKU. They used three different VBCDs (qDBS Capitainer®, Hemaxis‐D™ B10 and Neoteryx Mitra® device) in comparison with conventional DBS cards, with mean biases between the two methods ranging from −0.3 to 1.2% for Phe and from −0.5 to 1.6% for Tyr. The authors not only found that the three VBCDs were more accurate and precise than DBS sampling for the measurement of Phe and Tyr, but they were also less affected by blood volume and HCT. These findings suggest that VBCDs have the potential to improve the monitoring of Phe and Tyr levels in patients with PKU.^44^

PKU was also assessed in the study conducted by Gao et al.^43^ They performed an approach to validate a VAMS‐dried blood method for measuring Phe levels in PKU patients and compared its results to those obtained from traditional venous blood samples. The study included 24 PKU patients who participated in a Phase 2 clinical trial. Phe levels were then measured in both method samples using high‐performance liquid chromatography–tandem mass spectrometry (HPLC–MS/MS). The study evaluated the accuracy, precision, linearity, stability and matrix effects of the VAMS method compared with the conventional venous blood method. A difference was observed in both the Bland–Altman analysis (−4.25%) and the Passing‐Bablok analysis (−4.49%). However, this difference is considered clinically insignificant as it is within the acceptable range of 10–15% for monitoring blood Phe levels in PKU patients. The findings of this study suggest that the VAMS‐dried blood method is a reliable and convenient alternative to traditional venous blood collection for measuring Phe levels in PKU patients. This method may have the potential to improve patient compliance with blood monitoring and lead to better outcomes for PKU patients.^43^

Another study in newborns investigated the impact of preanalytical variables, such as gestational age, weight, gender and time of sampling, on metabolite concentration in DBS samples.^39^ The study included 610 reference DBS samples from healthy newborns to determine reference concentrations of amino acids, acylcarnitines and succinylacetone. There was no significant effect of these variables on the concentration of any metabolites. These findings suggest that the reference intervals for these metabolites can be reliably used for interpreting DBS metabolomics data in neonates. They serve as robust references for metabolic disease screening using LC–QqQ–MS detector, regardless of gender, weight or gestational age.^39^

A recent study provides valuable insights into the influence of maternal lifestyle factors, drug intake and supplements during pregnancy on the metabolic profile of newborns.^42^ The study included 109 mother–infant pairs and utilised NBS test to assess the impact of various factors such as smoking, alcohol consumption and physical activity. Maternal habits were evaluated through a questionnaire, and NBS results were collected from the Abruzzo regional NBS laboratory. Flow injection analysis tandem mass spectrometry (FIA–MS/MS) was employed to analyse amino acids and acylcarnitines (CAR) in DBSs. The finding revealed that maternal smoking during pregnancy was associated with significantly higher concentrations of CAR 14:2 and CAR 18:2 in NBS samples. Conversely, increased maternal physical activity was linked to higher concentrations of glutamine and glutamate in newborns. However, maternal intake of iodised salt in diet was significantly related to a decreased level of free carnitine. While further research with larger and more diverse metabolic assessment methods is necessary, the study underscores the impact of maternal lifestyle on the metabolic health of newborns and emphasises the importance of being mindful of potential false positives during NBS.^42^

In their study, Mak et al.^40^ validate a targeted metabolomics panel for second‐tier NBS, evaluating its performance in identifying infants with four specific metabolic disorders: glutaric aciduria type 1 (GA1), multiple acylcarnitine deficiencies (MACD), methylmalonic acidemia (MMA) and propionic acidemia. The research included 156 DBS samples from infants with confirmed diagnoses of GA1, MACD, MMA or propionic acidemia, along with 194 DBS samples from healthy control infants.^40^

Using a rapid and high‐throughput LC–MS/MS method with a 3 min runtime per sample and a triple‐quadrupole mass spectrometer, the targeted metabolomics panel quantified 20 metabolites, including 16 acylcarnitines, three amino acids and succinylacetone. The results demonstrated high sensitivity and specificity for each of the four disorders. For GA1, the sensitivity was 100%, and the specificity was 99.5%. For MACD, the sensitivity was 94.7%, and the specificity was 99.5%. For propionic acidemia, the sensitivity was 100%, and the specificity was 99.5%. These high sensitivity and specificity values underscore the accuracy of the panel in identifying affected infants, thereby minimising false positives. This precision is crucial for preventing unnecessary family stress, medical interventions and healthcare extra costs.^40^

Last, Kilgore et al.^41^ aimed to develop a universal second‐tier NBS LC–MS/MS method for amino acids, lysophosphatidylcholines and organic acids in DBS samples, addressing the limitations of current NBS methods. The method was validated using a panel of reference DBS samples and clinical DBS samples from infants with confirmed metabolic disorders. The developed LC–MS/MS method demonstrates high sensitivity, specificity and linearity for the quantification of those metabolites and was shown to be robust and reproducible with excellent intra‐ and inter‐day precision.^41^ A summary of the sample collection, sample preparation and analytical method used in the publications included can be found in Tables 2 and 3.

**TABLE 2:** Sample collection and preparation (2022–2023) – targeted endogenous metabolomics.

Entry	Samples (collection location)	Microsampling technology (manufacturer device)/(size/volume)	Microsampling extraction methods		References
Targeted metabolomics					
1	Capillary blood (*n* = 349) (finger prick)	DBS^®^ (N/S) (3 mm/N/S)	*pre‐extraction*: *extraction*: *re‐dissolved*:	Disc + **MeOH** spiked in with **IS** + shaking + centrifuge + dry in pure nitrogen gas flow at 50°C Derivatization with Acetyl chloride/1‐butanol (10:90) + dry in pure N_2_ gas flow Mixed with 80% ACN aqueous	Wu et al.^37^
2	Capillary blood (*n* = 707) (Heel prick)	DBS® (N/S) (5 mm × 2/N/S)	*Pre‐extraction*: *Re‐dissolved*:	Disc + MeOH spiked with IS + shaking in incubator + drying at 37°C under N_2_ flow Re‐dissolution in MeOH/H_2_O	Zhan *et al.* (2022)^38^
3	Venous blood (*n* = 1)	DBS (PerkinElmer‐226 filter paper) (3.2 mm/10 µL) DBS (PerkinElmer‐226 Bioanalysis RUO Card) (8 mm / 10 µL) DBS (HemaXis‐DB10) (7 mm/20 µL and 50 µL) VAMS (Neoteryx Mitra Device) (N/S/20 µL and 50 µL) qDBS (Capitainer‐qDBS device) (6 mm/20 µL and 50 µL)	*Extraction*:	Disc + 80% MeOH spiked with SIL + agitation + dilution fivefold	Carling *et al.* (2022)^44^
4	Capillary blood (*n* = 610) (heel pick)	DBS (Whatman™ 903 Filter Paper Card)	*Extraction*: *Storage*:	Dried and stored in biorepository (Clinical Laboratory Standards Institute guideline)	Jafri *et al.* (2023)^39^
		(N/S)			
5	Blood (*n* = 883) (NS)	DBS (N/S) (3.2 mm/N/A)	*Extraction*: *Re‐dissolved*:	MeOH:H_2_O:FA (80:20:0.1) + IS + vortex + centrifugation + dry with nitrogen gas flow Reconstitution with MeOH:H_2_O:FA (80:20:0.1)	Mak *et al.* (2023)^40^
6	Blood (*n* = NS)	DBS (Eastern Business Forms 903 Card) (3.2 mm/∼3.1 µL)	*Sample collection*: *Pre‐extraction*: *Extraction*:	Enrichment of blood with unlabelled biomarkers + spotted in filter paper cards Dry overnight + stored at −20°C Optimisation of sample preparation with MeOH:H_2_O and ACN:H_2_O in different concentrations with or without 0.1% FA, OX and IS	Kilgore *et al.* (2023)^41^
7	Blood (*n* = 109) (NS)	DBS (3.2 mm/∼3.2 µL)	*Sample collection*: *Extraction*:	DBS disc + extraction solution with IS (NeoBase 2 Non‐Derivated MSMS Kit) + agitation + incubation MeOH:H_2_O and ACN:H_2_O	Cicalini *et al.* (2023)^42^
8	Venous blood (*n* = 312) (NS)	VAMS^®^ (Neoteryx Mitra^®^ Device) (NA/20 µL)	*Sample collection*: *Pre‐extraction*: *Extraction*:	4 tips of VAMS clamshell collected Tip + IS working solution (0.1 FA in H_2_O) + Protein precipitation extraction with ACN:MeOH solution (50:50) + SN diluted with 0.1% FA in water	Gao *et al.* (2023)^43^

Abbreviations: CAN, acetonitrile; FA, formic acid; H_2_O, water; IPA, isopropanol; IS, internal standard; MeOH, methanol; N_2_, nitrogen; N/A, not apply; N/S, not specified; OX, oxalic acid; SIL, stable isotope label; SN, supernatant.

**TABLE 3:** Analytical methodology and instrumental analysis (2022–2023) – targeted endogenous metabolomics.

Entry	Separation technique	Ionisation/analysis mode	MS analyser	Standards	Analytical strategy and/or application in targeted metabolomics	References
Targeted lipidomics						
1	DI	ESI (+)/NS	QTRAP	Carnitine/acylcarnitine internal standard	Application of DBS to identify biomarkers of human gastric cancer Screening of significantly differential metabolites	Wu et al.^37^
2	RPLC C18	ESI (+)/MRM	Xevo QqQ	Testosterone‐d3, 4‐androstenedione‐d3, 11‐deoxycortisol‐d2, 17‐hydroxyprogesterone‐d8, cortisol‐d4	Method validation for targeted steroid profile Application of DBS to newborn screening for congenital adrenal hyperplasia in China	Zhan *et al.* (2022)^38^
3	FIA	ESI (+)/SRM	QqQ	Phenylalanine and tyrosine stable isotope (SIL)	Comparison between quantitative and non‐quantitative BµS devices Evaluation of HCT effect, volume test, bias between different collection device, imprecision and stability of Phe and Tyr on VAMS devices	Carling *et al.* (2022)^44^
4	LC (NS)	ESI/MRM	QqQ	Labelled internal standard (NS)	Evaluation of the time of sampling Application of DBS to evaluate the effect of preanalytical variables in neonates	Jafri *et al.* (2023)^39^
5	RPLC C18	ESI (+/−)/MRM	QqQ	Methylmalonic acid‐d3, labelled carnitine standards (NSK‐B‐1, NSK‐B‐G1), MSK‐A2 amino acids mix, ornithine ^13^C5, citrulline ^13^C5, dimethylglycine‐d6, uridine ^13^C5, orotic acid‐d2	Application of DBS for validating a targeted metabolomics in second‐tier newborn screening for inherited metabolic disorders	Mak *et al.* (2023)^40^
6	RPLC HILIC	ESI (+/−)/MRM	QqQ	LPC (20:0‐D4), LPC (22:0‐D4), LPC (24:0‐D4), LPC (26:0‐D4)	Method development of Second ‐ Tier of DBS for newborn screening Evaluation of lipid recovery and reproducibility	Kilgore *et al.* (2023)^41^
7	FIA	ESI (+)/MRM	QqQ	NeoBase 2 Non‐derivatised MSMS kit	Application of DBS for impact of maternal lifestyle in newborn metabolic profile	Cicalini *et al.* (2023)^42^
8	RPLC C18	ESI (+)/MRM	QqQ	2,3‐^13^C2,15N‐phenylalanine, ^13^C9, 15N‐phenylalanine	Validation of plasma and VAMs dried blood phenylalanine method by assessing selectivity, recovery, stability, inter and intra‐assay accuracy and precision	Gao *et al.* (2023)^43^

Abbreviations: DI, direct injection; ESI, electrospray ionisation; FIA, flow injection analysis; LPC, lysophosphatidylcholine; MRM, multiple reaction monitoring mode; NSK‐B‐G1, Labelled Carnitine Standards; NS, not specified; QqQ, triple quadrupole; QTRAP, quadrupole orbitrap mass spectrometer; RPLC, reverse phase liquid chromatography; SRM, selective reaction monitoring.

### 2.2 Exogenous molecules: TDM, anti‐doping, forensic, pollution

For the years 2022 and 2023, 29 articles were found using targeted metabolomics for exogenous molecules. Articles were found for TDM (18), anti‐doping (five), forensic studies (four) and per‐ and polyfluoroalkyl (PFAS) exposition (two).

A summary of the sample collection, sample preparation and analytical method used in the publications included can be found in Tables 4 and 5.

**TABLE 4:** Sample collection and preparation (2022–2023) – targeted exogenous metabolomics.

Entry	Samples (collection location)	Microsampling technology (manufacturer device)/(size/volume)	Microsampling extraction methods		References
Targeted metabolomics					
1	Capillary blood (finger prick) (*n* = 25)	VAMS (Mitra, Neoteryx) qDBS (Capitainer) (10 µL)	*Extraction*:	Tacrolimus: H_2_O, IS Creatinine: H_2_O, IS, cold ACN, H_2_O:MeOH (80:20)	Vethe et al.^46^
2	Capillary blood (finger prick) (*n* = 40)	VAMS (Mitra, Neoteryx) (20 µL)	*Pre‐extraction*: *Extraction*:	H_2_O MeOH:H_2_O (80:20), 40 mM zinc sulphate	Wang *et al.* (2022)^47^
3	Capillary blood (finger prick)	DBS (Whatman 903, Whatman Uni‐Core) (6 mm) VAMS (Mitra, Neoteryx) (20 µL)	*Extraction*: *Re‐dissolved*:	MeOH:H_2_O (80:20), IS H_2_O, 2 mM ammonium formate, 0.1% formic acid:ACN (60:40)	Paniagua‐González *et al.* (2022)^48^
4	Whole blood (*n* = 6) Capillary blood (finger prick) (*n* = 52 × 2 DBS × 2 VAMS)	DBS (Whatman 903) (3 mm) VAMS (Mitra, Neoteryx) (20 µL)	*Extraction*:	DBS: ACN, IS, zinc sulphate VAMS: ACN:H_2_O (40:60) ACN + IS	Mathew*et al.* (2022)^49^
5	Capillary blood (finger prick) (*n* = 40 × 2)	VAMS (Mitra, Neoteryx) (20 µL)	*Extraction*:	Tacrolimus: 50% MeOH, 0.1 M zinc sulphate, 100% ACN, IS Creatinine: 50% MeOH, 0.1 M zinc sulphate, 100% ACN, IS, H_2_O add to the supernatant	Scuderi *et al.* (2023)^50^
6	Capillary blood (finger prick) (*n* = 25)	DBS (Whatman 903) (8 mm) VAMS (Mitra, Neoteryx) (20 µL)	*Extraction*:	DBS: IS, 0.1 M zinc sulphate VAMS: IS, MeOH	Zwart *et al.* (2022)^51^
7	Whole blood (venous blood)	DBS (Whatman 903, Whatman DMPK‐C) (6 or 8 mm) Hemaxis DB10 (HemaXis) VAMS (Mitra, Neoteryx) qDBS (Capitainer) (10 µL)	*Extraction*:	Depend of the participating laboratory: MeOH:H_2_O MeOH:ACN ACN:H_2_O MeOH:EtOH H_2_O MeOH, zinc sulphate, ACN H_2_O then MeOH, 0.1 M zinc sulphate	Veenhof *et al.* (2023)^52^
8	Whole blood (venous blood)	qDBS (Capitainer) (10 µL)	*Extraction*:	H_2_O:MeOH (90:10) LLE: HCL + MTBE	Deprez *et al.* (2023)^53^
9	Whole blood (venous blood)	HemaPEN (Neoteryx) (2.74 µL)	*Extraction*:	MeOH:H2O (80:20), IS	Rosé *et al.* (2023)^54^
10	Capillary blood (finger prick) Whole blood (venous blood)	DBS (Whatman 903) (8 mm) Hemaxis DB10 (Hemaxis) (10 µL)	*Pre‐extraction*: *Extraction*:	IS H_2_O, formic acid, ammonium formate	Canil *et al.* (2023)^55^
11	Capillary blood (finger prick)	VAMS (Mitra, Neoteryx) (20 µL)	*Pre‐extraction*: *Extraction*: *Re‐dissolved*:	H_2_O ACN + IS MeOH:H_2_O (42:58)	Zimmermann *et al.* (2022)^56^
12	Whole blood (venous blood) (*n* = 10)	DBS (Whatman 31 ET CHR, Whatman 903) (8 mm)	*Extraction*:	MeOH, 0.1% formic acid	Zanchetta (2023)^57^
13	Whole blood (venous blood)	DBS (Whatman 903) (8 mm)	*Pre‐extraction*: *Extraction*: *Re‐dissolved*:	IS MeOH H_2_O, 5% acetic acid	Silva *et al.* (2023)^58^
14	Whole blood	qDBS (Capitainer) (10 µL)	*Extraction*:	MeOH, IS	Liu *et al.* (2023)^59^
15	Whole blood (venous blood)	DBS (Whatman 903) (10 mm)	*Extraction*: *Re‐dissolved*:	H_2_O, 2% formic acid, IS MeOH:H_2_O (50:50)	Xiaoyong *et al.* (2022)^60^
16	Whole blood (venous blood)	DBS (Whatman 903, Whatman DMPK‐B) (6 mm)	*Extraction*:	ACN:H_2_O (50:50), IS	Hu *et al.* (2023)^61^
17	Whole blood (venous blood)	DBS (Whatman 903) (6 mm)	*Extraction*:	ACN:H_2_O (90:10)	Herrera‐Pérez *et al.* (2023)^62^
18	Whole blood (venous blood) (*n* = 52)	VAMS (Mitra, Neoteryx) (10 µL)	*Extraction*:	H_2_O, MassTox^®^ Antimycotic Drugs/EXTENDED kit, IS	Simeoli *et al.* (2023)^63^
19	Whole blood (venous blood) (*n* = 6)	DBS (NS) (6 mm elution clamp)	*Extraction*:	MeOH:H_2_O (40:60), IS	Jing *et al.* (2022)^68^
20	Whole blood (venous blood) (*n* = 6)	DBS (Whatman DMPK‐C) (20 µL)	*Pre‐extraction*: *Extraction*: *Re‐dissolved*:	IS Cyclohexane:IPA:MeOH (60:20:20) Acetic acid:MeOH:H_2_O (30:60:10)	Yuan *et al.* (2022)^71^
21	Whole blood (venous blood) (*n* = 69)	DBS (NS) (6 mm)	*Extraction*: *Re‐dissolved*:	IS, MTBE:MeOH:IPA (50:25:25) MeOH:H_2_O:methoxyamine	Okano *et al.* (2022)^72^
22	Capillary blood (finger prick) (*n* = 129)	DBS (ShellCore security kit, developed by the China Anti‐Doping Agency (CHINADA)) (NS)	*Extraction*: *Re‐dissolved*:	H_2_O:IS, K2CO3:KHCO3, MeOH MTBE (LLE) Methoxylamine	Wang *et al.* (2022)^74^
23	Capillary blood (finger prick, upper arm) Whole blood (venous blood) (*n* = 10)	DBS (Whatman DMPK‐A, Whatman DMPK‐B, Whatman DMPK‐C) HemaSpot‐HF (SpotOnSciences) VAMS (Mitra, Neoteryx) Tasso M20 (Tasso) (20 µL)	*Extraction*: *Re‐dissolved*:	Depend of the device: MeOH (Mitra, Tasso M20); MeOH:ACN (50:50) or MeOH:IPA (50:50) (DBS cards, Hema‐Spot‐HF) H_2_O:ACN (95:5)	Mazzarino *et al.* (2022)^77^
24	Whole blood (*n* = 32)	Blood stains (cotton or plastic) (N/A)	*Extraction*:	ACN:H_2_O (60:40), 0.9% sodium chloride, IS	Adamowicz *et al.* (2022)^78^
25	Whole blood (venous) (*n* = 9)	DBS (QIAGEN QIAcard Bloodstain card (ThermoFischer)) (6 mm)	*Extraction*: *Re‐dissolved*:	MeOH, IS H_2_O:ACN (90:10)	Abarca *et al.* (2023)^82^
26	Whole blood (venous) (*n* = 105)	DBS (Whatman FTA) (10 mm)	*Extraction*:	MeOH	Guo *et al.* (2023)^84^
27	Whole blood (venous) (*n* = 7)	DBS (Whatman DMPK‐C) (30 µL)	*Extraction*: *Re‐dissolved*:	MeOH:ACN (75:25), IS MeOH	Massano *et al.* (2022)^85^
28	Whole blood (*n* = 6)	DBS (NS) (¼ of a 14 mm spot)	*Pre‐extraction*: *Extraction*: *Re‐dissolved*:	IS MeOH, 20 mM NaOH MeOH:H_2_O (50:50)	Lin *et al.* (2023)^87^
29	Whole blood (*n* = 18)				Rosen Vollmar *et al.* (2023)^90^

Abbreviations: ACN, acetonitrile; H_2_O, water; IPA, isopropanol; IS, internal standard; LLE, liquid–liquid extraction; MeOH, methanol; MTBE, methyl *tert*‐butyl ether; N/A, not apply; N/S, not specified.

**TABLE 5:** Analytical methodology and instrumental analysis (2022–2023) – targeted exogenous metabolomics.

Entry	Separation technique	Ionisation/analysis mode	MS analyser	Standards	Analytical strategy and/or application in targeted metabolomics	References
Targeted metabolomics						
1	Tacrolimus: LC–MS/MS Creatinine: FIA‐TQS	N/S	QqQ	^13^C‐tacrolimus‐d2, ^13^C3‐creatinine‐d3	TDM – immunosuppressant Monitoring of tacrolimus and creatinine	Vethe et al.^46^
2	Acquity UPLC BEH C18 (100 × 2.1 mm, 1.7 µm)	ESI (+)/MRM	QqQ	^13^C‐tacrolimus‐d4, mycophenolic acid‐d3, creatinine‐d3	TDM ‐–immunosuppressant Monitoring of tacrolimus and creatinine tacrolimus, mycophenolic acid and creatinine	Wang *et al.* (2022)^47^
3	ACQUITY UPLC BEH Shield RP18 (50 × 2.1 mm, 1.7 µm) + Acquity BEH Shield RP18 VanGuard column (5 × 2.1 mm, 1.7 µm)	UniSpray ion source (+)/MRM	QqQ	^13^C‐tacrolimus‐d2, mycophenolic acid‐d3	TDM – immunosuppressant Monitoring of tacrolimus and mycophenolic acid Comparison between DBS and VAMS	Paniagua‐González *et al.* (2022)^48^
4	Atlantis T3 C18 (100 × 2.1 mm, 3 µm)	ESI (+)/MRM	QqQ	Ascomycin, creatinine‐d3	TDM – immunosuppressant Monitoring of tacrolimus and creatinine	Mathew*et al.* (2022)^49^
5	Tacrolimus: Acquity HSS T3 (50 × 2.1 mm, 1.8 µm) Creatinine: Acquity BEH C18 (50 × 2.1 mm, 1.7 µm)	ESI (+)/MRM	QqQ	Tacrolimus‐d3, creatinine‐d3	TDM – immunosuppressant Monitoring of tacrolimus and creatinine	Scuderi *et al.* (2023)^50^
6	Acquity UPLC BEH C18 (50 × 2.1 mm, 1.7 µm) + Acquity UPLC BEH C18 (5 × 2.1 mm, 1.7 µm)	ESI (+)/MRM	QqQ	^13^C‐creatinine‐d3, ^13^C‐everolimus, ^13^C‐sirolimus‐d3, ^13^C‐tacrolimus‐d4, ^13^C‐mycophenolic acid‐d3, cyclosporine A‐d12, iohexol‐d5	TDM – immunosuppressant Monitoring of tacrolimus, mycophenolic acid, sirolimus, everolimus, cyclosporine, creatinine and iohexol	Zwart *et al.* (2022)^51^
7	Depend of the participating laboratory	Depend of the participating laboratory	Depend of the participating laboratory	Depend of the participating laboratory	TDM – immunosuppressant Monitoring of tacrolimus, ciclosporin, everolimus, sirolimus and mycophenolic acid Proficiency testing pilot between 14 laboratories	Veenhof *et al.* (2023)^52^
8	Acquity UPLC BEH C18 (50 × 2.1 mm, 1.7 µm) + Acquity UPLC BEH Amide VanGuard™ (5 × 2.1 mm, 1.7 µm)	ESI (+)/MRM	QqQ	Everolimus‐d4, ^13^C‐sirolimus‐d3, ^13^C‐tacrolimus‐d4, cyclosporin‐d12, creatinine,d3, ^13^C‐creatinine‐d3, ^13^C‐creatinine	TDM – immunosuppressant Monitoring of tacrolimus, sirolimus, everolimus, cyclosporin A and creatinine	Deprez *et al.* (2023)^53^
9	Luna Phenyl‐Hexyl (50 × 2 mm, 5 µm)	ESI (+)/MRM	QqQ	^13^C‐tacrolimus‐d2	TDM – immunosuppressant Monitoring of tacrolimus	Rosé *et al.* (2023)^54^
10	Cortecs T3 (75 × 3 mm, 2.7 µm) + VanGuard T3 (5 × 2.1 mm, 2.7 µm)	ESI (+)/MRM	QqQ	Olaparib‐d8, ^13^C‐rucaparib‐d3, ^13^C6‐niraparib hydrochloride	TDM – anti‐cancer Monitoring of 3 PARP Inhibitors	Canil *et al.* (2023)^55^
11	Waters XBridge Phenyl column (50 × 2.1 mm, 3.5 µm)	ESI (+)/MRM	QqQ	Afatinib‐d6, ^13^C1‐axitinib‐d3, bosutinib‐d9, cabozantinib‐d4, dabrafenib‐d9, lenvatinib‐d5, nilotinib‐d6, ^13^C1‐osimertinib‐d3, ruxolitinib‐d9, ^13^C6‐trametinib	TDM – anti‐cancer Monitoring of 10 kinase inhibitors	Zimmermann *et al.* (2022)^56^
12	Synergi™ Fusion‐RP column (30 × 2.0 mm, 4 µm) + Fusion‐RP Security Guard™ precolumn	Turbo IonSpray source (+)/MRM	Qtrap	Lenvatinib‐d4	TDM – anti‐cancer Monitoring of lenvatinib	Zanchetta (2023)^57^
13	Acquity UPLC HSS C18 (150 × 2.1 mm, 1.8 µm)	ESI (+)/MRM	QqQ	5‐Clorouracil	TDM – anti‐cancer Monitoring of 5‐fluorouracil	Silva *et al.* (2023)^58^
14	Acquity UPLC HSS T3 (50 × 2.1 mm, 1.8 µm)	ESI (+/−)/MRM	QqQ	Acetaminophen	TDM – antibiotics Monitoring of 8 antibiotics in neonates	Liu *et al.* (2023)^59^
15	Acquity UPLC HSS T3 (100 × 2.1 mm, 1.8 µm) + BEH C18 guard column	ESI (+)/MRM	QqQ	Norvancomycin, meropenem‐d6, linezolid‐d3	TDM – antibiotics Monitoring of 3 antibiotics in ill children	Xiaoyong *et al.* (2022)^60^
16	Kinetex XB‐C18 (50 × 2.1 mm, 1.7 µm)	ESI (+)/MRM	QqQ	Meropenem‐d6	TDM – antibiotics Monitoring of meropenem in preterm neonates	Hu *et al.* (2023)^61^
17	Acquity HSS T3 column (100 × 2.1 mm, 1.8 µm)	ESI (+)/MRM	QqQ	N/S	TDM – anti‐tuberculosis drugs Monitoring of 4 anti‐tuberculosis drugs	Herrera‐Pérez *et al.* (2023)^62^
18	LC (MassTox^®^ Antimycotic Drugs/EXTENDED kit)	ESI (+)/MRM	QqQ	MassTox^®^ Antimycotic Drugs/EXTENDED kit	TDM – anti‐fungal medication Monitoring of 3 anti‐fungal triazole agents in children	Simeoli *et al.* (2023)^63^
19	Zorbax Eclipse XDB‐C18 column (100 × 2.1 mm, 3.5 µm)	HESI (+)/PRM	Orbitrap	Testosterone propionate‐d3, testosterone decanoate‐d3	Anti‐doping Detection of anabolic steroid esters Automated online DBS sample preparation	Jing *et al.* (2022)^68^
20	Accucore XL C8 analytical column (100 × 3 mm, 4 µm)	HESI (+)/MRM	Orbitrap	Methenolone enanthate	Anti‐doping Detection of 20 endogenous anabolic steroid esters	Yuan *et al.* (2022)^71^
21	BEH C8 (50 × 2.1 mm, 1.7 µm)	ESI (+)/MRM	QqQ	N/S	Doping control during Olympic and Paralympic Games Detection of anabolic steroid esters	Okano *et al.* (2022)^72^
22	Hypersil GOLD (100 × 2.1 mm, 1.9 µm)	ESI (+)/PRM	Orbitrap	Testosterone‐d3, clenbuterol‐d9, mefruside	Doping control during Olympic and Paralympic Games Detection of anabolic steroid esters	Wang *et al.* (2022)^74^
23	Supelco Ascentis C18 column (150 × 2.1 mm, 2.7 µm)	ESI (+/−)/PMR	Orbitrap	Acetazolamide‐d3, amphetamine‐d11, bumetanide‐d5, cocaine‐d3, fluconazole‐d4, letrozole‐d4, morphine‐d3, triamcinolone acetonide‐d7, 17α‐Methyltestosterone, JWHO18‐d5	Anti‐doping Screening of 235 doping drugs and metabolites Comparison of volumetric and non‐volumetric devices	Mazzarino *et al.* (2022)^77^
24	Kinetex C18 (100 × 4.6 mm, 2.6 µm)	ESI (+)/dMRM	QqQ	Amphetamine‐d5	Forensic toxicology Quantification of drugs in crime scene bloodstains	Adamowicz *et al.* (2022)^78^
25	Agilent InfinityLab Poroshell 120, EC‐C18 (100 × 2.1 mm, 2.7 µm) + Agilent EC‐C18 guard (5 × 2.1 mm, 2.7 µm)	ESI (+)/MRM	QToF	Alpramazon‐d5, α‐hydroxyalprazolam‐d6, hydrocodone‐d6	Forensic toxicology Quantitation of alprazolam, α‐hydroxyalprazolam and hydrocodone	Abarca *et al.* (2023)^82^
26	Allure PFPP column (100 × 2.1 mm, 5 µm)	ESI (+)/MRM	QqQ	N/S	Forensic toxicology Detection of 425 drugs and poisons	Guo *et al.* (2023)^84^
27	Phenomenex Kinetex C18 column (100 × 2.1 mm, 1.7 µm)	ESI (+)/MRM	QToF	AH‐792‐d6, fentanyl‐d5, JWH‐018‐d9, JWH‐081‐d9, JWH‐250‐d5, mCPP‐d8, mephedrone‐d3, mescaline‐d9, MDPV‐d8, norfentanyl‐d5, Oxycodone‐d6, PCP‐d5, 251‐nBOMe‐d3	Forensic toxicology Detection of 132 psychoactive substance	Massano *et al.* (2022)^85^
28	Restek PFAS delay column (50 × 2.1 mm, 5 µm); Thermo Hypersil Gold C‐18 column (100 × 2.1 mm, 1.9 µm) + Accucore Q guard column (10 × 2.1 mm, 2.6 µm)	ESI (+)/MRM	Orbitrap	^13^C‐labelled PFAS	Trace analysis of 22 PFAS	Lin *et al.* (2023)^87^
29					Comparison between PFAS, thyroid hormone levels and neonatal characteristics	Rosen Vollmar *et al.* (2023)^90^

Abbreviations: ESI, electrospray ionisation; FIA, flow injection analysis; HESI, heat electrospray ionisation; MRM, multiple reaction monitoring mode; N/S, not specified; PMR, parallel reaction monitoring; QqQ, triple quadrupole; TDM, therapeutic drug monitoring; ToF, time of flight; UPLC, ultra performance liquid chromatography.

#### 2.2.1 Therapeutic drug monitoring

After using BµS to look for disease biomarkers, researchers naturally stayed in the health field and started to look for the therapeutic drugs, their behaviour and the response of the metabolome. TDM helps to know if the medication dosage is adapted to the patient in order to hold a constant level of the drug, for example, an immunosuppressant after transplantation.

BµS is not yet used in routine TDM workflow, but several studies show an increasing interest in this type of microsampling devices.^45^ Indeed, microsampling devices need a small amount of blood, most analytes are stable even at ambient temperature, and since the sample is dry it is less biohazardous than wet blood.^1^ Furthermore, since TDM requires repeated sampling in short intervals, BµS are of great interest. In the last 2 years, BµS has been studied in the monitoring of immunosuppressant drugs used after organ transplantation like tacrolimus and the associate biomarker creatinine,^45^, ^46^, ^47^, ^48^, ^49^, ^50^, ^51^, ^52^, ^53^, ^54^ anti‐cancer such as PARP and kinase inhibitors,^55^, ^56^, ^57^, ^58^ antibiotics in children and neonates,^59^, ^60^, ^61^ anti‐tuberculosis drugs^62^ and anti‐fungal medication.^63^

#### 2.2.2 Antidoping

Urine is the most used matrix for routine drug testing. Indeed, most doping substances’ metabolites are eliminated via the kidneys,^64^ and it is a quick and non‐invasive sample collection. Since 1992, blood has also been used in anti‐doping routines in complement of urine analysis.^65^ World Anti‐Doping Agency (WADA) and its accredited laboratories have been doing research on DBSs since 2000,^66^ and WADA is currently thinking about implementing DBSs into the Athlete Biological Passport.^67^ Indeed, BµS allowed to improve monitoring of blood hormone and drug levels and help to support the evaluation of circulating drug concentrations during a competition (doi.org/10.1080/10408363.2022.2103085).

Anabolic androgenic steroids (AASs) are used by athletes to increase their muscle mass, physical strength and sportive performance as well as to recover from injury.^68^, ^69^ Steroid esters are often used in doping since they can prolong the half‐life of exogenously ingested endogenous AASs.^70^ Sadly, the abuse of steroid esters is not detectable directly in urine. The current method uses gas chromatography combustion isotope ratio MS – which is time and sample‐consuming – and compares the results to the ‘Steroidal Athlete Biological Passport’ since the isotope ratio of natural and synthetic hormones are different.^71^

Steroid esters are not stable in whole blood because they can be hydrolysed. Thankfully, the hydrolases are inactivated once the blood dries,^71^ making DBSs the ideal matrix to analyse steroid esters directly.^72^ However, the volume of blood is small (<50 µL), and the polarity of steroid esters is low. Therefore, a derivatisation step is needed to increase the sensitivity.^68^, ^71^

During the XXXII Olympic Games and the XVI Paralympic Games in Tokyo, Japan, in 2021, DBSs were tested for steroid esters analysis. A total of 11 testosterone esters, nine nandrolone esters and six boldenone esters were investigated in 70 samples from different types of athletes (including aquatics, athletics, boxing, canoe, rowing, weightlifting and wrestling athletes). Since the WADA technical document for DBS analysis had not been approved before the game, 20 µL of whole blood was added to the DBS card in the laboratory instead of a real DBS sample collection by finger pricking. After drying, a punch of 6 mm was mixed with an IS solution, MTBE, isopropanol and methanol. The extracted solution was dried, and the residue was dissolved in methoxylamine and methanol. The sample was incubated before analysis with the LC–QqQ–MS.^72^

During the XXIV Olympic Winter Games and the XIII Paralympic Winter Games in Beijing, China, in 2022, DBS samples were collected from the fingertips of the athletes for the first time. A collection and security kit called ShellCore was developed for the occasion.^73^ Six testosterone esters were analysed using LC–HRMS.^74^ The feedback from athletes about DBS sample collection was positive, so WADA would like to increase this kind of sample collection in the future without replacing the traditional ones.^75^

The WADA‐accredited anti‐doping laboratory of Rome transposed their anti‐doping screening methods in urine^76^ to DBSs. A total of 235 compounds (225 drugs and 10 main metabolites) were detected in a 20 min UHPLC run. Also, seven microsampling devices were tested, including DBS cards, VAMS, HemaSpot™ HF and Tasso‐M20™.^77^

#### 2.2.3 Forensic

Blood is one of the most found biological traces on crime scenes. Lots of analysis can be done with it, including toxicology. In forensic studies, it can be interesting to develop analytical methods on DBSs. Indeed, in some cases, you cannot find people but their blood on the crime scene. It is usually collected a long time after the crime, and it can be dry. With those bloodstains, it is possible to know if the person was under the influence of drugs, alcohol, medication or other substances. Since the exact volume of bloodstains is unknown, the volume can be estimated thanks to the IS versus endogenous amino acids ratio.^78^ Since the concentration of the drugs will only be estimated, the results must be taken cautiously. Plus, if the subject has abnormal amino acid levels, which can occur in certain diseases, the drug concentration will be underestimated or overestimated. But it can still give an idea of the state of the person at the moment of the bleeding.

In the case of car accidents, the driver is often injured. Their blood can be found on their cloth or in the car, and the bloodstains analyses can tell the driver's psychomotor performance at the time of the accident.^78^ Licit and Illicit drugs as morphine, methamphetamine and nordazepam are stable in dry bloodstains for at least 6 months.^79^ Benzodiazepines are not stable in whole blood and are easily hydrolysed.^80^ In dry blood, the enzyme that catalyses the hydrolysis is deactivated.^81^ Alprazolam and α‐hydroxyalprazolam have been analysed after being stored for 17 years at room temperature.^82^

DBS cards can be used in forensics to stock samples to perform future analysis. Indeed, DBS cards need a small amount of blood, take less space than whole blood for storage (no need for a freezer) and can be stored for several years. Several analytes (like the one with an ester or amide group) are degraded in whole but not in dry blood, due to the inactivation of the enzyme that causes the hydrolysis.^80^, ^81^, ^83^ Plus, liquid blood is subjected to freeze thaw cycles that can degrade the analytes.^82^

A study showed that stored at room temperature with a desiccant, 33 drugs including sedative hypnotics, antiepileptic drugs and antipsychotic drugs could still be detected with LC–MS/MS after 3–5 years. Between 2016 and 2018, blood was collected from 105 drug poisoning cases and prepared as DBS cards. They were stored between 3 and 5 years at room temperature, protected from light and humidity in a sealed bag with a desiccant. The whole blood was analysed at the time of sample collection.^84^

Massano et al.^85^ developed a method that can detect 132 drugs (synthetic opioids, cathinones, hallucinogens and fentanyl) in less than 10 min. They were able to detect and quantify those substances with concentrations between 5 and 100 ng/mL.^85^

#### 2.2.4 Per‐ and polyfluoroalkyl exposition

Per‐ and polyfluoroalkyl substances (PFAS) have been largely used since the 1940s in several fields (chemical, electronic, photographic, military, aviation, agricultural, packaging and textile applications). Unfortunately, PFAS are hardly degradable, and therefore they have a high bioaccumulation level. Since the early 2000s in the USA, the concentration of PFAS in human blood has been tracked regularly because of their risk to human health.^86^, ^87^ Currently, the PFAS screening is done through venous blood. Lin et al.^87^ developed a method to analyse 22 PFAS in DBSs. Parallel to this method development, Koelmel et al.^88^ use untargeted screening to detect novel perfluoroalkyl substances and analogues. A total of 28 PFAS and 86 potential PFAS were detected with their fragmentation pattern.

It is known that during pregnancy, a mother shares nutrients with the foetus, but also chemical contaminants.^89^ In 2023, Rosen Vollmar et al.^90^ compared the level of thyroid hormone in newborns with their level of PFAS. Both thyroid hormone and PFAS analysis were made through DBSs, using the method developed by Lin et al.^87^ The same year, Taibl et al.^91^ studied the effect of prenatal PFAS exposition on the newborn metabolome via untargeted metabolomics. The PFAS analysis was conducted on the mother serum and the newborn metabolome on DBSs.

## 3 UNTARGETED METABOLOMICS

Untargeted metabolomics analyses employing BµS devices were used in several studies between 2022 and 2023. The studies included evaluations on sample storage as well as general development of the methodology and the identification of potential biomarkers associated with disorders in newborns and adults (for example, galactosemia, very long‐chain acylcarnitine dehydrogenase deficiency [VLCADD] and medium‐chain acyl‐coenzyme A dehydrogenase deficiency [MCADD]).

Most of the studies used DBSs, while only one study used VAMS.^12^ The DBS sample preparation and extraction used in most cases punching with a punch size of 3 mm – except for one study where 15 mm punches were used^91^ – and extraction was performed with different mixtures of water with methanol and/or ACN by vortexing, agitating/shaking and/or sonicating on a total time from 30 to 120 min. Most studies (seven) used LC–MS/MS (time of flight; ToF), and Ottosson et al.^7^ implemented trapped ion mobility separation ToF (timsToF). Three of the studies reported the use of an Orbitrap LC–MS/MS system,^40^, ^91^, ^92^ and furthermore, one study implemented direct injection high‐resolution MS.^93^ Tables 6 and 7 show a summary of the set‐ups used in the found studies.

**TABLE 6:** Sample collection and preparation (2022–2023) – untargeted metabolomics.

Entry	Samples (collection location)	Microsampling technology (manufacturer device)/(size/volume)	Microsampling extraction methods		References
1	VLCADD newborns (*n* = 15) Healthy controls (*n* = 15) (Heel prick)	DBS (Whatman™ 903 Filter Paper Card) (3 mm/30 µL)	*Sample collection*	*Sample collection*: Dripping blood on the cards	Sebaa et al.^96^
			*Extraction*:	*Extraction*: 250 µL H_2_O:ACN:MeOH (20:40:40) + 120 min agitation at room temperature + vacuum drying	
			*Re‐dissolved*:	*Re‐dissolved*: 50% A:B mobile phase	
2	GAL (*n* = 15) Healthy (*n* = 39)	DBS (N/S) (3 mm/30 µL)	*Sample collection*	Dripping blood on the cards	Alodaib *et al.* (2023)^97^
			*Extraction*:	H_2_O:ACN:MeOH (20:40:40) + 120 min agitation at room temperature 600 rpm + vacuum drying	
			*Re‐dissolved*:	50% A:B mobile phase	
3	Development (*n* = 1) Validation (*n* = 60) (Heel prick)	DBS (Whatman™ 903 Filter Paper Card) (3 mm/30 µL)	*Sample collection*	Venous blood in 2 mL EDTA‐K2 tubes and dripped on the cards	Guo *et al.* (2023)^13^
			*Extraction*:	150 µL H_2_O:ACN:MeOH (20:40:40) + 30 min agitation at room temperature at 750 rpm + freeze‐drying	
			*Re‐dissolved*:	ACN:H_2_O (25:73)	
4	Newborns (*n* = 200)	DBS (N/S) (3 mm/30 µL)	*Extraction*:	100 µL H_2_O:MeOH (20:80) + 45 min agitation at room temperature 450 rpm + drying with nitrogen	Ottoson *et al.* (2023)^7^
			*Re‐dissolved*:	Reconstitution solution + 15 min agitation at 600 rpm	
5	Pregnant (*n* = 100) Newborns (*n* = 100)	DBS (Whatman™ 903 Filter Paper Card) (N/S)	*Sample collection*	Collected in BD Vacutainer tubes with acid citrate dextrose (ACD) and transferred to the cards	Tobin *et al.* (2023)^92^
			*Extraction*:	N/S	
6	Newborns (*n* = 267) (Heel prick)	DBS (Guthrie card) (15 mm/70 µL)	*Extraction*:	1000 µL ice‐cold MeOH + 10 min vortex at 5000 rpm + 20 min ultrasound	Taibl *et al.* (2023)^91^
			*Re‐dissolved*:	H_2_O:MeOH (95:5)	
7	Patients (*n* = 49) Controls (*n* = 87)	DBS (Whatman™ 903 Filter Paper Card) (3 mm/30 µL)	*Extraction*:	N/S	Korteling *et al.* (2022)^93^
8	MCADD (*n* = 14) Controls (*n* = 14)	DBS (N/S) (3 mm/30 µL)	*Extraction*:	250 µL H_2_O:ACN:MeOH (20:40:40) + 120 min agitation at room temperature + vacuum drying	Sebaa *et al.* (2023)^95^
			*Re‐dissolved*:	50% A:B mobile phase	
9	Healthy (*n* = 10)	DBS (Whatman™ 903 Filter Paper Card) (3 mm/30 µL)	*Sample collection*	Venipuncture blood was collected and transferred to the cards	Schneider *et al.* (2023)^94^
			*Pre‐extraction*:	150 µL H_2_O:MeOH (50:50) + vortex and 15 min ultrasound	
			*Extraction*:	450 µL Ice‐cold MeOH:acetone (90:10) + 20 min agitation + vacuum drying	
			*Re‐dissolved*:	Reconstitution solvent + 15 min ultrasound	
10	Infants (*n* = 883)	DBS (N/S) (3 mm/30 µL)	*Extraction*:	200 µL MeOH:H_2_O (80:20) + 60 min Vortex + drying with nitrogen	Mak *et al.* (2023)^40^
			*Re‐dissolved*:	IS solution	
11	Women (*n* = 11) Men (*n* = 11)	VAMS (Neoteryx Mitra® Device) (N/S)	*Pre‐extraction*:	Hydration 5 s + 200 µL H_2_O:ACN (30:70) + 15 min ultrasound	Volani *et al.* (2023)^12^
			*Extraction*:	60 min vortex at 1200 rpm + vacuum concentrator	
			*Re‐dissolved*:	150 µL H_2_O:ACN (50:50), IS	

Abbreviations: ACN, acetonitrile; IS, internal standard; H_2_O, water; MeOH, methanol; N/S, not specified.

**TABLE 7:** Analytical methodology and instrumental analysis (2022–2023) – untargeted metabolomics.

Entry	Separation technique	Ionisation/ Analysis mode	MS analyser	Standards	Analytical strategy and/or application in untargeted metabolomics	References
1	Xselect (100 × 2.1 mm, 2.5 µm)	ESI (+/−)	qToF	N/S	To identify metabolic biomarkers and pathways altered in VLCADD neonates	Sebaa et al.^96^
2	Xselect HSS (100 × 2.1 mm, 2.5 µm)	ESI (+/−)	qToF	N/S	To identify metabolic biomarkers in patients with galactosemia	Alodaib *et al.* (2023)^97^
3	Waters BEH C8 (50 × 2.1 mm, 1.7 µm) ACQUITY UPLC HSS T3 column (50 × 2.1 mm, 1.8 µm)	ESI (+/−)	qToF	Combination of internal standards	To develop a DBS‐based metabolomics analysis method and demonstrate its analytical and clinical validation on a congenital hypothyroidism study with newborns	Guo *et al.* (2023)^13^
4	Acquity HSS T3 (100 × 2.1 mm, 1.8 µm)	ESI (+)	timsToF	N/A		Ottoson *et al.* (2023)^7^
5	BEH C18 (100 × 2.1 mm, 1.7 µm) BEH Amide (150 × 2.1 mm, 1.7 µm)	HESI‐II	Orbitrap	N/S	To identify profiles of preterm birth among pregnant women living with HIV on two different antiretroviral therapy regimens	Tobin *et al.* (2023)^92^
6	HSS T3 C18 (100 × 2.1 mm, 1.7 µm)	N/S	Orbitrap	L‐tryptophan‐d5	To profile the neonatal metabolome of maternal PFAS concentrations during early to middle pregnancy and gestational age at birth outcomes	Taibl *et al.* (2023)^91^
7	N/A	ESI	DI‐HRMS	N/A	To identify a metabolic ‘signature’ for 22q11.2DS and analyse the low intellectual functioning and autism spectrum disorder (ASD)	Korteling *et al.* (2022)^93^
8	XSelect (100 × 2.1 mm, 2.5 µm)	ESI (+/−)	qToF	N/A	To investigate potential metabolic biomarkers and pathways for MCADD newborns	Sebaa *et al.* (2023)^95^
9	Xselect HSS T3 XP (150 × 2.1 mm, 2.5 µm)	ESI	qToF	N/A	To profile the metabolomes of dried body fluid spots of blood, semen, saliva and urine over 4 weeks	Schneider *et al.* (2023)^94^
10	Two‐column coupling: HSS T3 column (50 × 2.1 mm, 1.8 µm) + AmazeHD column (50 × 2.1 mm, 10 µm)	ESI (+/−)	Orbitrap	Combination of internal standards	To develop an expanded metabolite panel for second‐tier testing of DBS samples for glutaric acidemia type I (GA1), methylmalonic acidemia (MMA), ornithine transcarbamylase deficiency (OTCD) and very long‐chain acyl‐CoA dehydrogenase deficiency (VLCADD)	Mak *et al.* (2023)^40^
11	Acquity BEH amide (100 × 2.1 mm, 1.7 µm)	ESI (+/−)	qToF	Combination of internal standards	To investigate the short‐term stability of VAMS samples and to evaluate differences in the metabolic profiles between VAMS‐sampled capillary blood to venous whole blood and venous whole blood‐derived plasma samples	Volani *et al.* (2023)^12^

Abbreviations: DI‐HRMS, direct infusion high‐resolution mass spectrometry; ESI, electrospray ionisation; FIA, flow injection analysis; HESI, heated electrospray ionisation; N/A, not apply; N/S, not specified; PNS, positive/negative ionisation switching; qToF, quadrupole time of flying mass spectrometer; timsToF, trapped ion mobility spectrometry–time of flying mass spectrometer.

The studies had different aims and settings, for example, optimisation of sample preparation or analysis of the stability of the specimens. Guo et al.^13^ compared the effect of applying different volumes of blood (35 or 75 µL) on the absorbing material by taking central and outer 3 mm punches and the coverage using 1, 2 or 3 blood punches. They found higher concentrations of some metabolites (e.g., 12 metabolites including carnitine, adenosine monophosphate, palmitoleic acid and other lipids) when sampling in the centre than outside when using 75 µL of blood, while a more homogenous distribution when using 35 µL. They interpreted that this was due to the chromatographic effects of the filter paper and a higher residence time with higher sample volumes. Furthermore, a single 3 mm punch reached more than 90% of the metabolite coverage of the three punches.^13^

Moreover, few studies tested the stability of blood micro samples.^7^, ^12^, ^13^ Guo et al.^13^ tested the stability for 1 year at 4 and −20°C, sampling at 4, 14 and 365 days. The samples presented acceptable stability at 4°C within just 4 days and at −20°C within 14 days but not anymore for 365 days.^13^ Ottosson et al.^7^ report on a long‐term stability study with neonatal DBS samples stored at −20°C for 10 years. They concluded that more than 71% of the DBS metabolome was stable,^7^ most of the unstable metabolites were affected either by the biobank or the transportation storage, and only a few metabolites were strongly influenced by storage. Volani et al.^12^ evaluated the short‐term stability of VAMS samples from 2 h to 2 weeks at room temperature and 4°C. They found that in the samples below 6 h, the metabolome varied less than 3%, from 1 day to 1 week 10% up to 60% and after 1 week of storage, the variations decreased.^12^ Overall, these results support the idea of storing DBSs at −80°C for long‐term storage.^13^ However, if there is no need to detect lipid metabolites, then long‐term storage at −20°C is a suitable option.^12^ Furthermore, samples should ‘rest’ in storage at −80°C to decrease variation if they are not collected at the same time, for example, in a longitudinal study.^12^ Complementary stability studies are needed to understand the temperature and storage effects on different metabolite groups in other types of blood micro samples.

Guo et al.^13^ also presented a validation of the analytical method evaluating reproducibility, linearity, the limit of detection and recovery. Complementary, they conducted a clinical validation on a metabolomics study of congenital hypothyroidism. The method had satisfactory stability, precision, linearity and recovery, with a limit of detection minimum from 0035 ng/mL for CAR 8:0‐d3 to 26 ng/mL for salicylic acid, a linear range between 3 and 5 orders of magnitude, relative standard deviations below 15% at different concentrations, and less than 30% in intra‐ and inter‐day replicates.^13^ When applying the method in the clinical trial, it allowed them to detect 249 metabolites qualitatively and quantitatively and to select 14 metabolites to differentiate between the control group and the patients with congenital hypothyroidism.^13^

Volani et al.^12^ additionally evaluated the qualitative and quantitative metabolic differences between samples from capillary and venous blood (whole blood and plasma) using VAMS. They found the largest overlap between capillary and whole blood, while the opposite for capillary blood and plasma. Quantitatively, metabolites detected in the three matrices had considerably different abundances between the matrices. Interestingly, the results for capillary samples presented the lowest reproducibility or larger variations. However, the authors still concluded that using VAMS with capillary blood samples can capture biological differences of interest not found in plasma samples.^12^

Conversely, Schneider et al.^94^ compared the use of DBSs with other body fluids and dried spots usually found in crime scenes aiming to find a universal biomarker and determine time since deposition. They peak‐picked 24,904 possible molecular features shared across all four body fluids. However, they concluded that each biofluid should be evaluated using independent biomarkers. From the blood‐specific compounds, they verified that four of them (l‐phenylalanyl‐l‐alanine, 3‐indole acrylic acid, spermidine and argininosuccinic acid) could be used to monitor time since deposition dynamics over 4 weeks.^94^

Two studies used untargeted metabolomics on DBSs to discover potential biomarkers for metabolic diseases associated with fatty acid β‐oxidation: very VLCADD and MCADD.^95^, ^96^ The authors compared DBS samples from newborns positively diagnosed with the specific disorder against healthy controls. The authors found distinctive metabolomics profiles, altered metabolic pathways and interesting potential metabolic biomarkers (3,4‐dihydroxytetradecanoylcarnitine, PIP (20:1/PGF1alpha), and PIP2 (16:0/22:3) for VLCADD newborns, and glutathione and oxidised lipids such as PGP (a‐21:0/PGF1alpha) in MCADD newborns). The results suggested that oxidative stress results from both diseases, increasing the concentration of glutathione as an antioxidant defense.^95^, ^96^ The same group also investigated patients with galactosemia, a genetic disorder related to disturbed galactose metabolism, and found alterations in the metabolome (e.g., PA (8:0/LTE4) and ganglioside GT1c (d18:0/20:0)) measured from DBSs.^97^ However, the study populations in all of these studies were small (*n* = 14–15) and therefore these results need to be validated in larger cohorts.

Taibl et al.^91^ used newborns' DBS untargeted metabolomics to explore the molecular mechanisms between prenatal exposure to four PFASs (perfluorohexane sulfonic acid, perfluorooctane sulfonic acid, perfluorooctanoic acid and perfluorononanoic acid) exposure and gestational age‐at‐birth outcomes. Metabolites linked to foetal PFAS exposure suggested alterations in redox homeostasis, tissue neogenesis and endocrine disruption. They could be responsible for the linked health consequences including reduced length of gestation.^91^ Furthermore, Tobin et al.^92^ also identified metabolic signatures associated with preterm birth from DBS collected from pregnant women with HIV during pregnancy and infants at birth. They analysed samples collected before antiretroviral initiation and during treatment with either zidovudine or a protease‐inhibitor‐based regimen. The authors suggest untargeted metabolomics as a tool for hypothesis generation and biomarker discovery in complex disease processes, based on potential compounds (urate, methionine sulfone, cortisone and 17α‐hydroxypregnanolone glucuronide) and mechanisms found as potentially linked with preterm births.^92^

Korteling et al.^93^ used untargeted metabolomics to analyse DBS samples of children and adolescents with a syndrome associated with increased risk of neurodevelopmental phenotypes including autism spectrum disorders (ASD) and intellectual impairment. The untargeted analysis identified metabolic signatures (the authors highlighted proline as a prominent distinguishing biomarker) and the metabolites were associated with the phenotypic expressions of ASD and intelligence quotient impairment.^93^

Overall, in these clinical investigations of metabolome using DBSs, untargeted metabolomics enabled the identification of novel potential biomarkers to differentiate patients from healthy controls. These results also increase the understanding of the diseases and the involved biological pathways. However, most of the studies were cross‐sectional and relatively small. There is a need for more longitudinal studies with larger cohorts.

## 4 LIPIDOMICS

The association between BµS strategies and lipidomic analysis is still in its early stages of development, and there are not many research studies on their use and applications in this field, but they have become a promising method for less invasive blood collection means that they are gradually being considered as a promising and favourable method for lipidomic studies.^31^, ^36^, ^98^

Among the most relevant articles published, between 2022 and 2023, we have selected seven papers where six use LC–MS methods and 1 uses FIA–MS method. In this section, the information has been organised in Tables 8 and 9, which summarise the research studies from a methodological point of view, focusing on the device used, sample collection, preparation, instrumental analysis and also the main goals and applications of the selected lipidomic studies. Some of the BµS techniques already summarised in Table 1 have also been gradually contributing to the field of lipidomics, as noted in the previous reviews.^31^, ^36^, ^98^

**TABLE 8:** Sample collection and preparation (2022–2023) – lipidomics.

Entry	Samples (collection location)	Microsampling technology (manufacturer device)/(size/volume)	Microsampling lipid extraction methods		References
Targeted lipidomics					
1	Capillary blood (*n* = 34) (finger prick)	VAMS^®^ (Neoteryx Mitra^®^ Device) (N/A/30 µL)	*pre‐extraction*	*Pre‐extraction*: Mitra tip + MeOH spiked in with IS + vortex	Shen et al.^8^
			*extraction*:	*Extraction*: MTBE + incubation under agitation at 4°C + ice cold H_2_O addition + vortex + centrifugation + organic phase dry under N_2_	
			*re‐dissolved*: *storage*:	*Re‐dissolved*: MeOH	
2	Capillary blood (*n* = 19) (finger prick)	DBS (hemaPEN^®^ Device) (3.5 mm/4 × 2.74 µL)	*sample collection*: *extraction*:	Blood is collected through four EDTA‐coated capillaries, then the tube is placed upside down and blood is transferred onto pre‐punched Whatman paper discs via gravity action. MeOH:H_2_O (80:20) spiked in with IS + sonication + homogenisation + centrifugation + paper disc removal + evaporation at 30°C in a centriVAP concentrator	Laurent *et al.* (2023)^34^
			*Re‐dissolved*:	22% MeOH:EtOH (50:50) + 78% ACN:H_2_O (95:5) + 0.1 M AA & 0.1% FA	
3	Deuterated LPC standard mixture	DBS (Eastern Business Forms 903 Card) (NS/100 µL)	*extraction*: *re‐dissolved*:	ACN:H_2_O (80:20) + 0.1% FA + 1 mM OX) spiked in with IS + 8 mg/mL DTT in H_2_O + dry under N_2_ ACN:H_2_O (90:10), 0.18% FA, 4 mM AF, 66.6 µM OX + shaking	Kilgore *et al.* (2023)^41^
4	Capillary blood (*n* = 10) (finger prick)	qDBS (Capitainer^®^ B Collection Device) (6 mm/10 µL)	*Extraction*:	IPA:MeOH (50:50) + IS + 20 ceramic balls + sonication + centrifugation + supernatant evaporation	Meikopoulos *et al.* (2023)^14^
			*Re‐dissolved*:	IPA:MeOH (50:50)	
Untargeted => targeted lipidomics					
5	Whole blood (N/S) (venous)	DBS (Whatman™ 903 Filter Paper Card) (6 mm/3.2 µL) PSC (Noviplex™ DUO Plasma Prep Card) (6 mm/3.2 µL)	*Sample collection*: *Extraction*:	75 µL of K _2_ N/S	Bishop *et al.* (2023)^104^
Untargeted lipidomics					
6	Capillary blood (*n* = 12) (armprick)	DBS (Tasso‐M20™ Device) (N/S/4 × 1.75 µL)	*Extraction*:	IPA:MeOH (50:50) + sonication + incubation at 4°C + centrifugation + upper phase isolation	Cendali *et al.* (2023)^35^
7	Whole blood (*n* = 24) (heal/finger prick)	DBS (PerkinElmer^®^ 226 Five Spot RUO Card) (5 × 3.2 mm/N/S)	*Extraction*: *Storage*: *Re‐dissolved*:	H_2_O + DCM:MeOH (33:66) + vortex + incubation at 4°C + vortex + H_2_O + DCM + organic phase dry under N_2_ DCM + IS+ IPA:MeOH (50:50)	Ferreira *et al.* (2023)^103^

Abbreviations: AA, ammonium acetate; ACN, acetonitrile; AF, ammonium formate; DCM, dichloromethane; DTT, DL‐dithiothreitol; EtOH, ethanol; FA, formic acid; H_2_O, water; IPA, isopropanol; IS, internal standard; MeOH, methanol; MTBE, methyl‐*tert‐*butyl ether; OX:, oxalic acid; N_2_, nitrogen; N/A, not apply; N/S, not specified. LPC, lysophosphatidylcholine; PSC, Telimmune Plasma Separation Cards.

**TABLE 9:** Analytical methodology and instrumental analysis (2022–2023) – lipidomics.

Entry	Separation technique	Ionisation/analysis mode	MS analyser	Standards	Analytical strategy and/or application in lipidomics	References
Targeted lipidomics						
1	N/A FIA	PNS/MRM	QTRAP	SCIEX Lipidyzer™ Platform kits (50 IS)	Stability of lipids in VAMS Quantitative comparison between VAMS vs. venous blood Application of VAMS in lifestyle context	Shen et al.^8^
2	RPLC C18	ESI(+/−)/dMRM	QqQ	LPC(18:1‐d7), Sphingosine‐d7	Application of hemaPEN in lipidomic studies related to physical effort	Laurent *et al.* (2023)^34^
3	RPLC HILIC	ESI(+/−)/MRM	QqQ	LPC(20:0‐d4), LPC(22:0‐d4), LPC(24:0‐d4), LPC(26:0‐d4)	Method development of second‐tier of DBS for newborn screening Evaluation of lipid recovery and reproducibility	Kilgore *et al.* (2023)^41^
4	RPLC C18	ESI(+)/MRM	QqQ	Deuterated Ceramide LIPIDOMIX^®^ Mass Spec Standard	Method development for ceramides identification from qDBS Quantitative comparison of qDBS vs. venous blood	Meikopoulos *et al.* (2023)^14^
Untargeted => targeted lipidomics						
5	RPLC C18	ESI(+/−)/DDA MS/MS	Hybrid Q‐Orbitrap	For recovery evaluation: UltimateSPLASH™ ONE decanoyl‐l‐carnitine‐d3, dodecanoyl‐l‐carnitine‐d3, octadecanoyl‐l‐carnitine‐d3, palmitic acid‐d3, oleic acid‐d9, arachidonic acid‐d11, cholesterol‐d7, C17‐sphingosine	Evaluation of lipid recovery and storage stability in PSC Validation PSCs for untargeted lipidomic profiling Quantitative comparison of PSC vs. DBS	Bishop *et al.* (2023)^104^
Untargeted lipidomics						
6	RPLC polar lipids: C18 non‐polar: ACQUITY HSS T3	ESI(+/−)/MS/MS	Orbitrap	N/S	Application of DBS to demonstrate lipidomic changes during physical effort^a^	Cendali *et al.* (2023)^35^
7	RPLC C18	HESI(+/−)/DDA MS/MS	Orbitrap	PC(14:0/14:0), PE(14:0/14:0), PG(14:0/14:0), PI(16:0/16:0), PS(14:0/14:0), PA(14:0/14:0), LPC(19:0), SM(d18:1/17:0), Cer(d18:1/17:0) CL(14:0/14:0/14:0/14:0)	Application of DBS to demonstrate lipidomic changes at different paediatric ages^b^	Ferreira *et al.* (2023)^103^

Abbreviations: N/A, not apply; FIA, flow injection analysis; PNS, positive/negative ionisation switching; MRM, multiple reaction monitoring; QTRAP, quadrupole orbitrap mass spectrometer; RPLC, reverse phase liquid chromatography; ESI, electrospray ionisation; dMRM, ring; QqQ, triple quadrupole; HESI, heated electrospray ionisation; N/S, not specified; PSC, Telimmune Plasma Separation Cards.

^a^ A total of 156 relevant molecular lipids, from 11 lipid subclasses, reported and identified using MS‐DIAL 4 software tool based on MS/MS spectra.

^b^ A total of 118 relevant molecular lipids reported (N/S) and identified using LipidSearch 4.0 (Thermo Scientific) software tool based on accurate intact mass, isotopic pattern and fragmentation pattern.

The selected articles of the last year are in line with previous lipidomic studies,^99^, ^100^, ^101^, ^102^ where the collection of DBSs on filter paper remains the most used approach today in lipidomic studies.^41^, ^103^, ^104^ When analysing these DBS card samples, it is assumed that the blood and analytes spread homogeneously. However, it is important to mention that DBS has a matrix effect dependent on the type of lipid and can cause interference in extraction efficiency and stability and contribute to ion suppression in LC–MS analysis.^105^ Another problem is the potential adsorption and non‐specific binding of lipids to the microsampling card, given that they contain cellulose with many hydroxyl groups that can interact with the hydrophilic head of the lipid molecules.^106^, ^107^

Alternatively, with advances in commercially available microsampling devices, Table 1, common barriers such as volumetric inaccuracies and the haematocrit effect in DBS microsampling can be overcome in lipidomics as well. Thus, several research studies evaluate the possibility of conducting reliable – both targeted and untargeted lipidomic – studies using hemaPEN^®^,^34^ Tasso‐M20™^35^ or qDBS Capitainer^®^.^14^ These devices, in addition to previous studies using VAMS,^100^, ^108^ allow the accurate collection of blood samples for comparison between traditional and advanced techniques^104^ (entry 5, Table 8) as well as comparison between microsampling and venous blood collection^8^, ^14^ (entries 1 and 4, Table 8). It demonstrates the great interest of these devices to collect samples very easily on the field by the athletes themselves by using low invasive techniques and requiring less organisation.

### 4.1 BµS lipid extraction methods, applied approaches and instrumentation

Sample collection and preparation is probably the most critical step to ensure reliable lipidomic data collection, and only a few studies have examined the influence of lipids storage stability, recovery and reproducibility.^8^, ^41^ To date, the evidence collected in Table 8 shows that there is no standardised method for lipid extraction from the different microsampling techniques. The extraction method depends on the type of lipid species being studied. For example, studies until 2021 agree that MeOH is the best solvent to extract phospholipids and fatty acids from DBSs.^36^ Traditionally and still today, solvent lipid extraction protocols use biphasic extraction method where lipids are commonly extracted using mixtures of water and organic solvents (mostly MTBE, CH_3_Cl and MeOH).^8^, ^34^, ^41^, ^103^ During the last few years, attempts have been made for reducing extract complexity using single‐step extraction protocols; either with two solvent combination: methanol/isopropanol or butanol mixtures – or three solvent combination such as MeOH/MTBE/CH_3_Cl mixtures.^14^, ^35^ Although these last extraction methods has been evaluated and found to be best – for isolating lipids from plasma giving benefits from high coverage and simplified operation^109^ – more studies are needed to determine the best solvent system so that the extraction efficiency/recovery is maximised.

During sample preparation it is also common to include a drying period either by evaporation or under N_2_ action, followed by a re‐dissolution. Only a few recent studies investigate^8^, ^41^, ^104^ the appropriate and reproducible recovery of every class of lipids, the pH, temperature, requirement of solvation energy (shaking vs. sonication) and duration of the extraction process from DBSs, and this makes appropriate validation experiments necessary. ISs are commonly added, at appropriate concentrations, before the sample extraction to normalise the extraction efficiency and instrumental response (entries 1, 2, 3 and 4) or IS spiked into the extraction solvent (entries 1, 2 and 3). Stable isotope‐labelled lipids are routinely used as well as commercial mixtures of deuterated standards solutions such as Avanti deuterated ceramide LIPIDOMIX®^14^ or UltimateSPLASH™ ONE^104^ (Table 9).

From the seven lipidomic studies gathered in this review, there is a marked preference for targeted lipidomics analysis since it is clearly the most used approach (Table 9). The targeted approach aims to unravel specific pathways or profile‐specific markers, identifying and quantifying specific lipids. From the reviewed studies, RPLC–MS with a triple quadrupole (QqQ) analyser is the most common method, and only one study^8^ used FIA with a QTRAP analyser. Since we notice a current challenge in addressing the untargeted lipidomics approach, we will focus on this point.

### 4.2 Untargeted lipidomics

Only three lipidomic BµS studies have been found in the last year to demonstrate lipidomic changes during physical effort^35^ or at different paediatric ages.^103^ and to evaluate the lipid recovery and sample storage stability in plasma separation cards (PSCs) versus DBSs.^104^

Cendali et al.^35^ (entry 6, Tables 8 and 9) have applied RP–HPLC coupled to MS/MS with an orbitrap to demonstrate the utility of advanced BµS, in this case, Tasso‐M20™ Device to characterise metabolomic and lipidomic changes during exercise. For data analysis, an in‐house standard compound library was used, and lipidomic data were analysed using LipidSearch 4.0 (Thermo Scientific), identifying a total of 118 lipids. in this study, the hierarchical clustering of the top 50 significant lipids demonstrates a clear sex‐independent pattern of lipid depletion.^35^

On the other hand, Ferreira et al.^103^ (entry 7, Tables 8 and 9) assessed the variability of the lipid profile of DBSs at paediatric ages and identified age‐related variations using RP–HPLC–MS/MS. The data were identified using MS‐DIAL and integrated using MZmine v2.42 software, allowing the identification of 156 different lipid species that belong to 11 different classes. They have found that the lipidomic signature of the three age groups (0–10 days, 2–18 months and 3–13 years) is significantly different, and the study on this profile variability in children may help to improve the knowledge of the evolution of lipid metabolism in childhood, concluding that DBSs and lipidomics can be further explored.^103^

Finally, Bishop and Fiehn^104^ (entry 5, Tables 8 and 9) performed untargeted lipidomics to evaluate the lipid recovery and sample storage stability in PSCs; they also validated it for untargeted lipidomic profiling and made a quantitative comparison of PSCs versus DBSs, specifically (Noviplex™ DUO Plasma Prep Card and Whatman™ 903 Filter Paper Card). Although details for the sample extraction protocol are missing, they mention the use of RP–HPLC coupled to MS/MS using a hybrid Q‐Orbitrap. They used the open‐source software MS‐DIAL for deconvolution, peak picking, alignment and compound identification. Also, annotated compounds were done by matching retention times, accurate precursor masses, and MS/MS fragmentation patterns against the LipidBlast library, last MS‐FLO software was used to identify ion adducts, duplicate peaks and isotopic features. They identified 489 unique lipids, with 237 matching the criteria; in ESI positive ion mode, 357 lipid species and 67 labelled IS, and for ESI negative ion mode, 306 known compounds and 54 labelled IS. Overall, they found PSCs provide a better alternative for quantitative blood lipidomics analyses compared with DBSs.^104^

Great efforts have been made in this review to extract and unify the information from the articles found for lipidomic and microsampling. In this direction, the Lipidomics Standards Initiative (LSI) aims to standardise the reporting of lipidomics data and analyses and the terminology used.

## 5 SUMMARY AND OUTLOOK

Interest in BµS continues increasing as it is a convenient option for analysis in vulnerable groups and for recurrent or remote sampling; examples of this are NBS and sports monitoring and many others as demonstrated in this article. The reviewed metabolomic and lipidomic studies in 2022 and 2023 show that some of them are focused on the optimisation of the analytical workflow, verifying parameters such as recovery, reproducibility and storage stability, while other studies employed BµS for disease biomarkers identification and profiling, biological pathways understanding, developing screening methods and monitoring physical effort. In the context of target metabolites, specific applications are related to TDM, anti‐doping, forensic studies and PFAS exposure.

Despite the advantages of volumetric new technologies and the limitations of DBSs, such as the haematocrit effect, DBS was shown to be the preferred BµS in the studies, Mitra**^®^** (VAMS) as the second preferred, and just a few studies evaluated the potential of other quantitative devices in comparison with DBSs, plasma or whole blood.^14^, ^34^, ^35^, ^44^ A further and deeper investigation of the developed technologies is required to promote their acceptance from research‐related and non‐diagnosis activities to their implementation as a clinical or patient‐centred tool. Furthermore, several new devices have entered the market and will be tested for metabolomics and lipidomics applications. The collected results revealed, as previous reviews stated,^98^ that more efforts are needed to standardise and harmonise results across LC–MS and to avoid significant errors in analysis and reporting. To contribute to this goal, we encourage related scientists to (i) be more specific about the description of the analytical workflow conditions, as many experimental methods focused more on the clinical and biological characteristics (ii) implement into the analysis the use of QA (quality assurance) and QC (quality control) to register changes in the analysis over time, caused by retention‐time shift, degradation of the extracts or column and ion source contamination^110^ and (iii) consider the integration of ISs and standard reference materials to support the accuracy and reliability of the results and (iv) continue to further stages targeting longitudinal studies with larger cohorts.

The information obtained from the reviewed lipidomic studies show its implications in both basic biology and clinical medicine, as well as informing therapeutic strategies and improving health outcomes. The collected results have revealed, as previous reviews have already stated,^98^ that more efforts are needed to standardise and harmonise results across LC–MS lipidomic platforms and avoid significant errors in analysis and reporting. Not only the increased implementation of ISs but also of standard reference materials in analytical workflows will aid in this direction. Also, actual literature of microsampling reveals that there is a need for integrate into the analysis the use of QA (quality assurance) and QC (quality control) to register changes in the instrument sensitivity over time, caused by retention‐time shift, degradation of the extracts or column and ion source contamination by non‐volatiles.^110^ Great efforts have been made in this review to extract and unify the information from the articles found for lipidomic and microsampling. In this direction, the LSI (https://lipidomicstandards.org/) aims to standardise the reporting of lipidomics data and analyses, and the terminology used.

In conclusion, different BµS have shown great promise for application in targeted and non‐targeted metabolomics and lipidomics analysis. A recurring topic is the limited sample material that will be obtained by BµS. Certain analysis still requires a sufficient amount for sample preparation and pre‐concentration to achieve the required limits‐of‐detection. However, LC–MS/MS system sensitivity is increasing as well, and developments such as the switch from analytical flow to microflow also lead to higher sensitivity. Additionally, derivatisation procedures can also help to increase the sensitivity. Analytical scientists are asked to develop new and robust analysis methods. Likewise, automation of sample preparation will become an important issue. While automated sample preparation of liquid samples such as plasma and serum is well advanced and even used in targeted and non‐targeted metabolomics studies, this needs to be improved for BµS. Different automated systems have been developed, for example, an automated DBS sampler.^111^ VAMS can be used with 96‐well‐adapter for easier handling.

## CONFLICT OF INTEREST STATEMENT

The authors declare no conflicts of interest.
